# Persistence of a Yeast-Based (*Hanseniaspora uvarum*) Attract-and-Kill Formulation against *Drosophila suzukii* on Grape Leaves

**DOI:** 10.3390/insects11110810

**Published:** 2020-11-18

**Authors:** Flavia Bianchi, Urban Spitaler, Irene Castellan, Carlo S. Cossu, Timothy Brigadoi, Claire Duménil, Sergio Angeli, Peter Robatscher, Rudi F. Vogel, Silvia Schmidt, Daniela Eisenstecken

**Affiliations:** 1 Laboratory for Flavours and Metabolites, Institute for Agricultural Chemistry and Food Quality, Laimburg Research Centre, Laimburg 6, 39040 Auer (Ora), South Tyrol, Italy; flavia.bianchi@laimburg.it (F.B.); timothy.brigadoi1997@gmail.com (T.B.); peter.robatscher@laimburg.it (P.R.); 2 Chair of Technical Microbiology, TUM School of Life Sciences, Technical University of Munich, Gregor-Mendel-Straße 4, 85354 Freising, Germany; rudi.vogel@wzw.tum.de; 3 Entomology Group, Institute for Plant Health, Laimburg Research Centre, Laimburg 6, 39040 Auer (Ora), South Tyrol, Italy; urban.spitaler@laimburg.it (U.S.); Carlo-Simone.Cossu@laimburg.it (C.S.C.); Silvia.Schmidt@laimburg.it (S.S.); 4 Department of Crop Sciences, Institute of Plant Protection, University of Natural Resources and Life Sciences, Gregor-Mendel-Straße 33, 1180 Vienna, Austria; 5 Faculty of Science and Technology, Free University of Bozen-Bolzano, 39100 Bolzano, Italy; irene.castellan@natec.unibz.it (I.C.); claire.dumenil@unibz.it (C.D.); Sergio.Angeli@unibz.it (S.A.)

**Keywords:** spotted wing drosophila, pest control, spinosad, metabolites, VOCs

## Abstract

**Simple Summary:**

*Drosophila suzukii* is an invasive pest species that feeds on yeast-laden fruits and is attracted to fermentation products. In nature, numerous yeast species are associated with *Drosophila suzukii*. Yeasts constitute a food source and produce volatile compounds attractive to the fly. The production of attractants and chemical compounds that stimulate feeding by *Drosophila suzukii* make the use of yeasts promising for the development of attract-and-kill formulations. In the present work, the efficacy and the persistence over a one-week period of a yeast-based attract-and-kill formulation was evaluated treating grape plants in a greenhouse. The efficacy was assessed by measuring the survival and oviposition rate of *Drosophila suzukii*. The concentrations or presence/absence of potential feeding stimulants and attractants were assessed by quantitative measurement of carbohydrates, sugar alcohols, amino acids, and volatile compounds. Results show that the formulation was still effective and that some of the chemical compounds monitored were still present on the surface of treated leaves one week after treatment, though changes in the chemical profiles were observed over this period.

**Abstract:**

The production of phagostimulant and attractive volatile organic compounds (VOCs) by yeasts can be exploited to improve the efficacy of attract-and-kill formulations against the spotted wing drosophila (SWD). This study evaluated the persistence over one week of a yeast-based formulation under greenhouse conditions. Potted grape plants were treated with: (i) potato dextrose broth (PDB), (ii) PDB containing spinosad (PDB + S), and (iii) *H. uvarum* fermentation broth grown on PDB containing spinosad (H. u. + S). Laboratory trials were performed to determine the survival and the oviposition rate of SWD after exposure to treated leaves. Ion-exchange chromatography was performed to measure carbohydrates, sugar alcohols, and organic acids on leaf surfaces, while amino acids were assessed through liquid chromatography–mass-spectrometry. Additionally, the VOCs released by plants treated with *H.uvarum* were collected via closed-loop-stripping analysis and compared to those emitted by untreated leaves. A higher mortality was observed for adult SWDs in contact with *H. uvarum* containing spinosad compared to PDB containing spinosad. Generally, a decrease in the amounts of non-volatile compounds was observed over time, though numerous nutrients were still present one week after treatment. The application of the yeast-based formulation induced the emission of VOCs by the treated leaves. The concentration of 2-phenylethanol, one of the main VOCs emitted by yeasts, decreased over time. These findings describe the presence of potential phagostimulants and compounds attractive to SWD in a yeast-based attract-and-kill formulation and demonstrate the efficacy of the formulation over one week.

## 1. Introduction

The spotted wing drosophila (SWD), *Drosophila suzukii* (Matsumura) is an important insect pest with a wide host range, including small soft fruit, stone fruit, and grapes [1,2]. The control of SWD in fruit cultivation relies usually on sprays of synthetic insecticides to reduce yield losses associated to SWD infestations [3]. Unfortunately, most insecticides are not selective and are facing severe limitations regarding pesticide residues in worldwide exports [4]. Yeasts were found to enhance the efficacy of insecticide treatments leading to a lower amount of insecticide needed to achieve a sufficient protection against the pest [5,6]. The possibility to exploit nutritional behavior and attractiveness induced by yeasts associated to SWD makes the use of these microorganisms a promising strategy for controlling SWD infestations [6,7,8,9,10]. One limit concerning the use of live microorganisms for control strategies is related to the microbial metabolic changes that occur in response to nutritional sources available in the medium [11]. Moreover, changes in the medium composition that occur during yeast growth and fermentation are reflected both in the loss of potentially phagostimulant compounds for SWD flies [12,13] and in modifications of the profile of volatile compounds [13,14], affecting the attractiveness to SWD [15]. For the development of an efficient attract-and-kill formulation, it is, therefore, important not only to select the appropriate species and growth medium that enhance the feeding stimulation and the attractiveness but also to ensure that the efficacy is maintained over time.

The present study aims at evaluating the persistence over time of a yeast-based attract-and-kill liquid formulation on grape plants in a greenhouse. *Hanseniaspora uvarum* was selected for the study because, among the numerous yeast species isolated from fruits infested by SWD, *Hanseniaspora* was reported as the predominant genus [16,17,18,19]. Additionally, *H. uvarum* was found to be more attractive to SWD adults and larvae in choice tests compared to other yeast species [20,21], as well as to promote the ingestion by SWD adults [12]. For the attract-and-kill formulation, spinosad was chosen as insecticide since it is allowed in viticulture, as well as in organic production, and is known to be very effective in toxic baits [6,10,22]. Grapevine leaves were treated with the yeast-based attract-and-kill formulation in a greenhouse. Mortality and oviposition assays were performed in the laboratory to evaluate the effects of the treatments on SWD adults over a one-week period. The persistence of yeast’s metabolites and nutritional compounds on the leaves’ surfaces, as well as volatile organic compounds (VOCs) emitted by the plants, were measured to evaluate their changes over time.

## 2. Materials and Methods

### 2.1. Insect Rearing

The SWD flies originated from different fruits in South Tyrol, Italy. The SWD flies were reared in a mass rearing on *Drosophila suzukii* cornmeal diet (DSCD(a) with dry deactivated yeast) and dry baker’s yeast (RUF Lebensmittelwerk KG, Quakenbrück, Germany) sprinkled on the surface on which they fed and laid the eggs [18]. The rearing contained 5% sucrose solution on cotton as additional sugar and water source. The SWD larvae developed on the cornmeal diet. Female and male SWD adults emerging from the pupal stage within three days were kept on cornmeal diet and sucrose solution in an insect cage (BugDorm—1, MegaView Science Co., Ltd., Taichung, Taiwan). When all flies were between five and eight days old, 20 female and 20 male SWD flies were placed together into one insect cage. Males were distinguished from females by the dark spot on the leading edge near the tip of each wing [1]. The insect cages were kept in climatic chambers at 22 ± 1 °C, with 65 ± 5% relative humidity and a photoperiod of L16:D8.

### 2.2. Yeast Cultivation

The yeast *Hanseniaspora uvarum* (strain: LB-NB-2.2, accession number GenBank NCBI: MK567898) was isolated from feeding tunnels of SWD larvae in infested grapes in South Tyrol, Italy [18]. Yeast cultures were grown under sterile conditions in 220 mL autoclaved potato dextrose broth (PDB; 4 g/L potato starch, 20 g/L dextrose, Difco^TM^, Becton Dickinson, Le Pont de Claix, France) at 25 °C, 120 rpm for 30 h under light in a 250-mL Erlenmeyer flask closed with cotton and aluminum foil. The inoculum (0.5 mL) was prepared with a loop full of yeast cells cultivated on potato dextrose agar (4 g/L potato starch, 20 g/L dextrose, 15 g/L agar, Difco^TM^, Becton Dickinson), which were transferred in a 2-mL Eppendorf tube filled with 1 mL PDB and vortexed for 10 s at 1800 rpm. After 30 h of growth, the yeasts reached the stationary phase. The number of cells per mL (Fuchs Rosenthal counting chamber, Assistent^®^, Sondheim vor der Rhön, Germany) was 6.4 × 10^7^, optical density (OD) at 600 nm (Cary 60 UV-Vis, Agilent, Santa Clara, CA, United States) was 1.8, and the pH (pH meter, Crison GLP 21, Hach, Düsseldorf, Germany) of the fermentation broth was 4.13. The yeast fermentation broths and autoclaved PDB were stored at −80 °C and thawed at room temperature before use.

### 2.3. Grape Plants Cultivation

Rooted grafted vines of the local variety “Edelvernatsch Lb 43” on rootstock SO4 were potted in 4-L pots filled with standard soil (SP ED63 T coarsely, Einheitserde^®^, Sinntal-Altengronau, Germany). The plants were grown for two months in the greenhouse and treated once a week for 20 min against powdery mildew with vaporized sulfur using a sulfur burner. No sulfur treatments were performed during the experimental period. The temperature and the relative humidity in the greenhouse were monitored over the experimental period (Table S1).

### 2.4. Treatments

**Figure 1:**
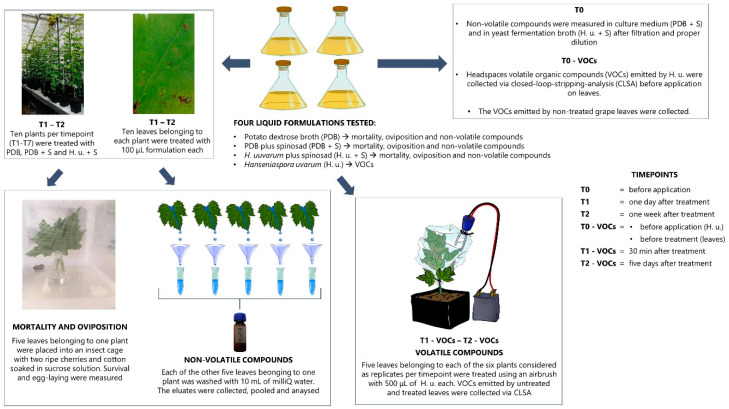
Experimental design of the treatments on potted grape plants in the greenhouse. A scheme of the assays and analyses performed is reported.

A summary of the experimental design is reported in Figure 1. Three different treatments were performed on grape leaves in the greenhouse for further laboratory trials of mortality and oviposition and for the analyses of non-volatile compounds: (i) insecticide-free PDB (PDB), (ii) insecticide-containing PDB (PDB + S), and (iii) insecticide-containing *H. uvarum* fermentation broth (H. u. + S). For the insecticide-containing samples, 11.32 µL/L Laser^TM^ (480 g/L spinosad, Corteva Agriscience^TM^, Milan, Italy) were added to *H. uvarum* fermentation broth or PDB with a resulting active ingredient (AI) of 5.43 mg spinosad per L. Each of the three different treatments were applied to ten plants at the same time. Ten leaves per plant were treated with 10 drops of 10 µL each using a multichannel pipette (Eppendorf Research Plus, Hamburg, Germany). Leaves belonging to five plants per treatment were ripped off one day after treatment (T1), while leaves belonging to other five plants were ripped off one week after treatment (T2). All treated leaves belonging to each of the five plants per timepoint were ripped off and used for further SWD assays and chemical analyses. For mortality and oviposition assays and analyses of non-volatile compounds, the same plants were used (five leaves belonging to one plant were used for assays and five for chemical analyses). Single plants were considered as replicates.

Since the amount of 10 drops was not sufficient for the detection of VOCs, for volatiles collection a slightly different treatment was performed. Six plants were treated with *H. uvarum* (H. u.), and each plant was considered as a replicate. Five leaves belonging to one plant were treated with 500 μL per leaf using an airbrush (Hansa 681, Harder & Steenbeck, Norderstedt, Germany) to cover the upper surface. The volatile collections were performed one day before treatment (T0—VOCs), as soon as the formulation dried on the leaves surface (ca. 30 min after treatment) (T1—VOCs) and five days after treatment (T2—VOCs). The VOCs emitted by six untreated plants were collected and considered as a control.

### 2.5. Mortality and Oviposition Assays

Five leaves belonging to the same plant were refreshed in an Erlenmeyer flask filled with tap water and closed with cotton to avoid the contact of the flies with water. The leaves were placed into the insect cage together with a small Petri dish (diameter 6 cm) containing cotton soaked in 10 mL of a 5% sucrose solution and two ripe cherries for oviposition. After 24 h and 48 h, the mortality of males and females was assessed, and the total number of eggs laid per cage was counted. The cherries were replaced by new cherries after 24 h. Single cages were used as replicates (*n* = 5). The insect cages were kept in climatic chambers at 22 ± 1 °C, with 65 ± 5% relative humidity and a photoperiod of L16:D8. The mortality was calculated as the total mortality over 24 h and 48 h and the oviposition as eggs laid during the first and the second 24 h.

### 2.6. Sample Preparation and Analysis of Chemical and Metabolic Compounds

For the analysis of metabolites, LC-MS grade solvents and reagents were used (VWR International Srl, Milan, Italy). Analytical standards of compounds under investigation were used for the quantitative analysis, and isotope-labeled internal standards (IS) of DL-Phenylalanine-3,3-d2, L-Lysine-4,4,5,5-d4 hydrochloride, L-Glutamic acid-2,3,3,4,4-d5, L-Alanine-2,3,3,3-d4 (Sigma-Aldrich, Merck KGaA, Darmstadt, Germany) were spiked into each sample for the analysis of amino acids.

Each leaf was washed with 10 mL of MilliQ water. The eluate from five leaves belonging to one plant were pooled and filtered (hydrophilic surfactant-free cellulose acetate filters, 0.2 µm). Single plants were used as replicates (*n* = 5). For carbohydrates and sugar alcohols, 1 mL of sample was transferred in a high performance liquid chromatography (HPLC) vial and analyzed with high-performance anion-exchange chromatography with pulsed amperometric detection (HPAE-PAD, Dionex ICS 5000, Thermo Fisher, Waltham, MA, United States). For organic acids, 500 µL of sample were freeze dried, resuspended in 100 µL of MilliQ water and analyzed using high-performance anion-exchange chromatography with conductivity detection (HPAE-CD, Dionex ICS 5000, Thermo Fisher, Waltham, MA, United States). For amino acids, 500 µL of sample were transferred in a HPLC vial containing 480 µL of acetonitrile and 20 µL of IS amino acids mix (50 mg/L). Samples were analyzed in liquid chromatography electrospray ionization triple quadrupole mass spectrometry (UHPLC-QqQ, Dionex UltiMate 3000 UHPLC TSQ Quantiva, Thermo Fisher, Waltham, MA, United States) in multiple reaction monitoring (MRM) mode. The liquid formulations were analyzed before their application on leaves (T0) after filtration and proper dilution. The analytical methods used were based on Spitaler et al. [12].

### 2.7. Volatile Compounds Collection and Characterization by CLSA-GC-MS

The VOCs were collected via closed-loop-stripping analysis (CLSA) and analyzed in gas chromatography–mass spectrometry (GC 7890A coupled with a MS 5975C Network, Agilent Technologies, Santa Clara, CA, USA). To reduce variation in chemical profiles due to the plant circadian rhythm, all collections were performed at a regular time between 12 p.m. and 3 p.m. The treated shoots were not covered during the five days between the first and the second collection. Untreated leaves were considered as a control. Plant materials were held in a VOC-bag (Cuki^®^ oven bag, Cuki Cofresco S.r.l., Volpiano, Italy). Charcoal filtered air was pushed in via an inlet port at a rate of 500 mL/min while air was sucked out via an outlet port at a rate of 400 mL/min, creating a positive pressure in the bag for three hours. The airflow was maintained using a 12 V graphite vacuum pump (Fürgut, Tannheim, Germany) using Teflon tubes and ferrule connections. The outlet air passed through an adsorbent trap (glass tube, 6.5 × 0.55 × 0.26 cm) loaded with 1.5 mg activated charcoal (CLSA filter LR-type; Brechbühler AG, Schlieren, Switzerland). The VOCs were eluted from the adsorbent traps with 100 μL GC-grade dichloromethane (Sigma-Aldrich, Merck KGaA, Darmstadt, Germany) and stored at −80 °C. Adsorbents were cleaned after each collection using three rinses with approximately 50 μL of HPLC-grade heptane, HPLC-grade methanol then GC-grade dichloromethane (all Sigma-Aldrich, Merck KGaA, Darmstadt, Germany) and baked 10 min at 160 °C. Two μL of extract were injected on a non-polar HP-5MS column (30 m × 0.25 mm ID, 0.25 μm film thickness, 7890A, Agilent Technologies, Santa Clara, CA, United States) in splitless mode when the inlet valve was at 280 °C. Helium was used as carrier gas at a flow rate of 1.2 mL/min and a velocity of 39.92 cm/s. The starting temperature of 50 °C was held for 1.5 min, followed by an increase of 7.5 °C/min until a temperature of 250 °C was reached and then held for 10 min.

VOCs emitted by the yeast fermentation broth were also collected via CLSA before their application on leaves (T0—VOCs). A double airflow pump system was used: charcoal-filtered air was pushed at rate of 1 L/min into a 250-mL Pyrex glass bottle containing 100 mL of yeast sample; simultaneously, CLSA filters (1.5 mg activated charcoal, LR-type, Brechbühler AG, Schlieren, Switzerland) fitted into the plastic lid of the glass bottle were connected to the outflow pump using a short Teflon tube, drawing out air at a rate of 0.4 L/min. The CLSA filters were then eluted with 100 µL of GC-grade dichloromethane solvent (Sigma-Aldrich, Merck KGaA, Darmstadt, Germany) in 1.1-mL GC glass vials (Sigma-Aldrich, Merck KGaA, Darmstadt, Germany) and stored in a freezer at −80 °C until use for subsequent GC-MS analysis.

The chromatogram was recorded in the full scan mode m/z 20–400 amu, and the electron ionization was set at 70 eV and the ion source temperature at 250 °C. Data acquisition and analysis were carried out using ChemStation software (Agilent Technologies, Santa Clara, CA, United States). A commercially available mixture of n-alkane standards (nC8-nC40, Sigma-Aldrich, Merck KGaA, Darmstadt, Germany) was used to calculate the linear retention indices (LRI) [23]. Compounds were annotated initially by comparing their mass spectra with those in the databases NIST 14 (Gaithersburg, MD, USA) and Wiley7 (Wiley, Hoboken, NJ, USA). The identity of all compounds, with the exception of 1,8-cineole and trans-alpha-bergamotene, was confirmed by comparison with reference standards (Sigma-Aldrich, Merck KGaA, Darmstadt, Germany) (Table 1).

### 2.8. Statistical Analysis

Statistical analyses were performed using the software R [24]. The mortality and oviposition data were analyzed with a one-way ANOVA. The equality of error variance was verified with a Levene’s test. Multiple comparisons were performed with Bonferroni’s procedure. To evaluate the variation of the concentration of each metabolite over time, a one-way ANOVA was applied using Tukey’s post hoc test for pairwise comparison. Nonparametric tests (Wilcoxon statistic with Bonferroni’s correction to adjust the significance level) were performed whenever at least one of the conditions to apply parametrical tests (normal distribution, variance homogeneity) was not satisfied. To evaluate if there was any significant difference between VOCs emitted by treated and non-treated leaves at two timepoints, all the compounds peak area means were compared using one-way ANOVA followed by post hoc Tukey’s test. The distributions of data and residuals were verified graphically with the functions qqp and qplot from the R package ggplot2 [25]. Statistical significance was assessed at the level of *p* < 0.05.

## 3. Results and Discussion

### 3.1. Mortality and Oviposition Assessment One Day after Treatment (T1)

**Figure 2:**
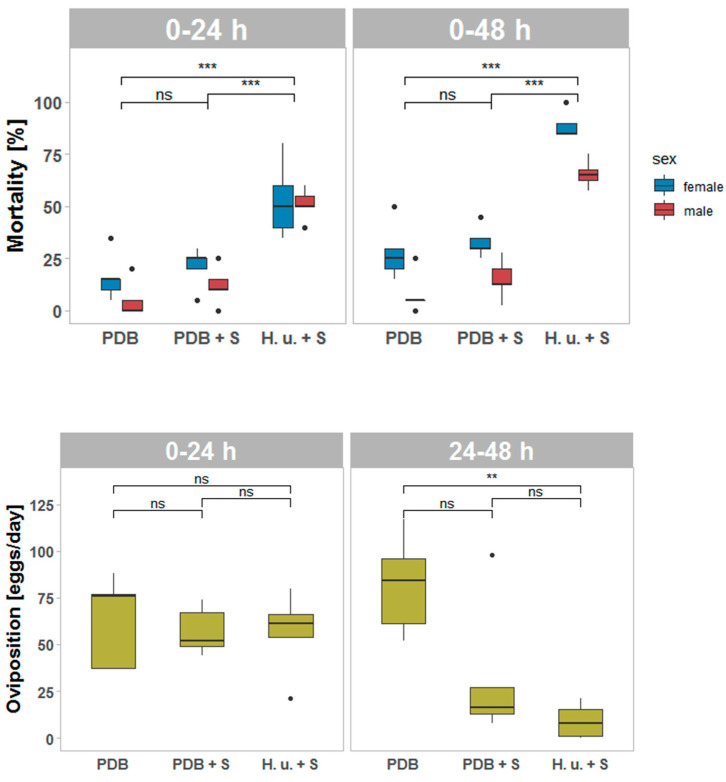
Mortality and oviposition (*n* = 5) of spotted wing drosophila (SWD) adults (20 males and 20 females) after the exposure to leaves treated with potato dextrose broth (PDB), PDB plus spinosad (PDB + S), or *H. uvarum* plus spinosad (H. u. + S) one day after treatment (T1). Mortality of males and females after 24 h and after 48 h of exposure. Oviposition after the first 24 h and between 24 and 48 h of exposure. Asterisks indicate significant differences between the treatments (*p* < 0.05). Not significant differences are reported, as well (ns). Outliers are indicated with dots. ≤0.01 = **; ≤0.001 = ***.

One day after treatment (T1), the mortality and the oviposition were affected by the different treatments (Figure 2). After 24 h (F_2, 7.673_ = 35.481, *p* < 0.001) and after 48 h (F_2, 12_ = 122.00, *p* < 0.001), a significant effect on the mortality of SWD flies was observed. Significantly more SWD died after exposure to H. u. + S compared with PDB or PDB + S (*p* < 0.05). This result confirms that the presence of yeast metabolites and VOCs is necessary to achieve a higher mortality rate compared to PDB + S. Within the first 24 h of exposure, the mortality of SWD flies in contact with H. u. + S lead to a 5.0-fold higher mortality compared to spinosad-free PDB. After 48 h of exposure, the treatment H. u. + S lead to a 4.3-fold higher mortality compared to PDB. Interestingly, no significant differences were observed between PDB and PDB + S (*p* < 0.05). This was surprising, since PDB contains glucose and sugars which were described as feeding stimulants for SWD in baits in combination with insecticides [7,26]. However, the absence of attractive fermentation products in PDB may prevent SWD flies from finding the insecticidal bait on the leaves. A similar ingestion by SWD females when fed with PDB and *H. uvarum* was observed in a previous study, where attractiveness did not play any role [12]. In this study, the flies had the possibility to feed on an additional sucrose solution in the cage and to move around in larger experimental cages. Therefore, based on the experimental setting, the flies could avoid contact with the insecticidal bait, meaning that emitted volatiles together with feeding stimulant compounds present in the yeast fermentation broth enhance the efficacy of the bait.

After 24 h of exposure to the treated leaves, the different formulations did not influence the oviposition (F_2, 12_ = 0.158, *p* = 0.855). Females of SWD oviposit eggs before reaching 5 days post-closure [27], and, when the fecund SWD flies entered the assay, some females immediately reached the fruits before getting in contact with the leaves. It was already shown that the mating status of SWD females influences the preference between fruits and fermentation volatiles. Seven-day-old mated females prefer strawberries, while virgin females prefer apple cider vinegar [28]. In the field, SWD females collected on fruit have more mature eggs in the ovaries than those collected in traps with fermentation baits [29]. The flies entered the assays five days after hatching and were already able to lay eggs. Therefore, it can be assumed that the females were able to lay eggs into the fruits present in the cages and that VOCs emitted by the fruits were attractive for the females before they get attracted by the VOCs emitted by yeasts. During the first 24 h, the females had time to get in contact with the bait. Therefore, a significant effect of the treatment became visible after the first 24 h (F_2, 12_ = 9.621, *p* = 0.003). Since oviposition was counted as the number of eggs laid per cage, these values reflect the influence of female mortality. A higher oviposition was observed comparing PDB treatment with PDB + S or H. u. + S (*p* = 0.05). No differences were observed between H. u. + S and PDB + S (*p* < 0.05). Even though there was no significant effect on the mortality comparing PDB and PDB + S, the lower oviposition caused by spinosad in the PDB + S treatment compared to PDB could have been due to the contact of some flies with a sublethal dose of spinosad (Figure 2).

### 3.2. Mortality and Oviposition Assessment One Week after Treatment (T2)

**Figure 3:**
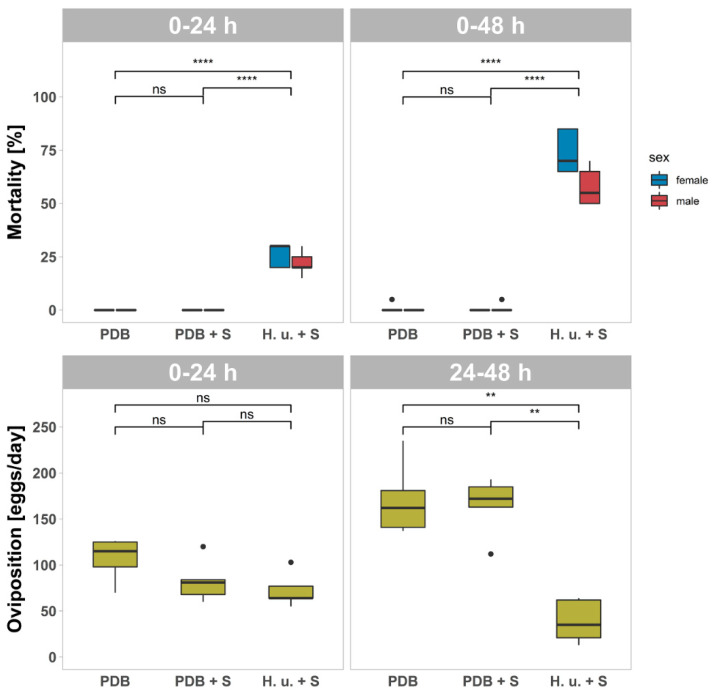
Mortality and Oviposition (±SD; *n* = 5) of SWD adults (20 males and 20 females) after the exposure to leaves treated with PDB, PDB plus spinosad (PDB + S), or *H. uvarum* plus spinosad (H. u. + S) one week after treatment (T2). Mortality of males and females after 24 h and after 48 h of exposure. Oviposition after the first 24 h and between 24 and 48 h of exposure. Asterisks indicate significant differences between the treatments (*p* < 0.05). Not significant differences are reported, as well (ns). Outliers are indicated with dots. ≤0.01 = **; ≤0.0001 = ****.

The same methodology used for T1 was used for T2 to evaluate the effects after one week (Figure 3). A significant effect of the formulations on the mortality after 24 h (F_2, 6.190_ = 694.376, *p* < 0.001) and after 48 h (F_2, 7.151_ = 131.912, *p* < 0.001) of exposure to the treated leaves was observed. As observed at T1, there was also no significant difference in the mortality comparing flies exposed to PDB and PDB + S (*p* < 0.05) at T2, while H. u. + S lead to a significantly higher mortality compared to the previous two treatments (*p* < 0.05). No differences were observed in the number of eggs laid in the first 24 h (F_2, 12_ = 3.239, *p* = 0.075) as for T1. Over the second 24 h of exposure, the influence of the formulations on the oviposition was significant (F_2, 12_ = 26.609, *p* < 0.001). Lower oviposition was observed with the treatment H. u. + S compared to PDB or PDB + S (*p* < 0.05).

### 3.3. Carbohydrates and Sugar Alcohols

**Figure 4:**
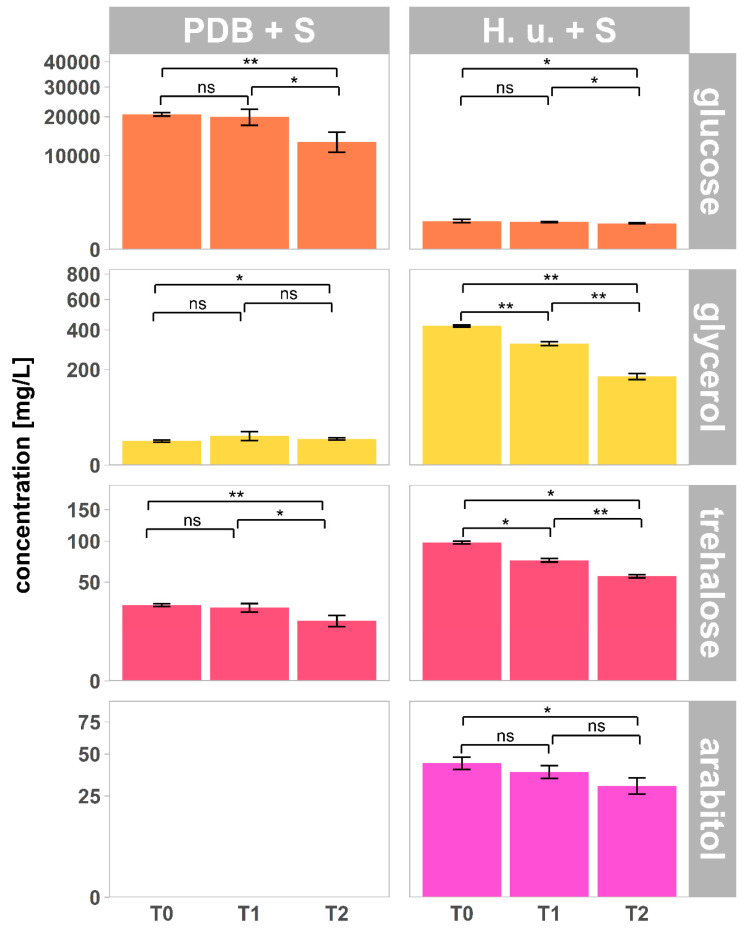
Concentration of glucose and sugar alcohols (mean ± SD; *n* = 5) in the culture broth PDB + S (T0), in the fermentation broth H. u. + S (T0) and on the surface of leaves treated with PDB + S and H. u. + S collected one day (T1) and one week (T2) after treatment. Arabitol was not detected at any of the timepoints in PDB + S. Asterisks indicate significant differences between timepoints for each treatment (*p* < 0.05). Not significant differences are reported, as well (ns). <0.05 = *; ≤0.01 = **.

Two carbohydrates (glucose and trehalose) and two sugar alcohols (glycerol and arabitol) were found in the analyzed samples. The amount of carbohydrates and sugar alcohols in culture and fermentation broths (T0), as well as on leaves treated with PDB + S and H. u. + S (T1 and T2), are reported in Figure 4. The concentrations and the trend over time of the chemical compounds analyzed on leaves treated with insecticide-free PDB were very similar to those of leaves treated with PDB + S; therefore, the results of insecticide-free PDB treatment were not reported in the figures. Yeasts consumed glucose and produced sugar alcohols and trehalose. The concentration of glucose on the leaves treated with PDB + S and H. u. + S significantly decreased over time (*p* < 0.05). This reduction in the sugar’s concentration, more evident in PDB + S but significant for both PDB + S and H. u. + S, may be a result of its biodegradation by epiphytic microorganisms populating the leaves’ surface. As for glucose, the concentrations of trehalose and sugar alcohols significantly decreased over time (*p* < 0.05), except for glycerol in PDB + S (Figure 4). Exogenous trehalose is a carbon source for bacteria [30]; thus, biodegradation of this compound can occur. Lactic acid bacteria populate the grape surface, and many of them are able to use diverse sugars and sugar alcohols as substrate [31]. The rapid biodegradation, coupled with a photodegradation half-life of 6.8 h [32], explains the rapid reduction of the concentration of glycerol on the leaf surface within one day of exposure to light.

Although a large amount of glucose was consumed by yeasts within 30 h of growth prior to the treatments, carbon sources suitable for SWD were still available one week after treatment. Concentrations of 173 mg/L glycerol, 30 mg/L arabitol, 56 mg/L trehalose, and 777 mg/L glucose were found on the surface of leaves treated with H. u. + S. Carbohydrates are known feeding stimulants in SWD flies [33]. Additionally, *Drosophila* flies possess gustatory receptors for trehalose [34], and this compound was found to elicit a response of sugar neurons [35]. Behavioral assays based on the proboscis extension reflex demonstrated that *Drosophila* flies extend proboscis to feed on glucose, trehalose, and glycerol [36], with the gene Gr64e conferring responsiveness to glycerol in *Drosophila* [37,38]. The lower amount of glucose in the yeast fermentation broth compared to PDB may, therefore, not necessarily be a limiting factor for the feeding acceptance of the attract-and-kill formulation by SWD, as long as it is coupled with the presence of sugar alcohols.

### 3.4. Amino Acids

**Figure 5:**
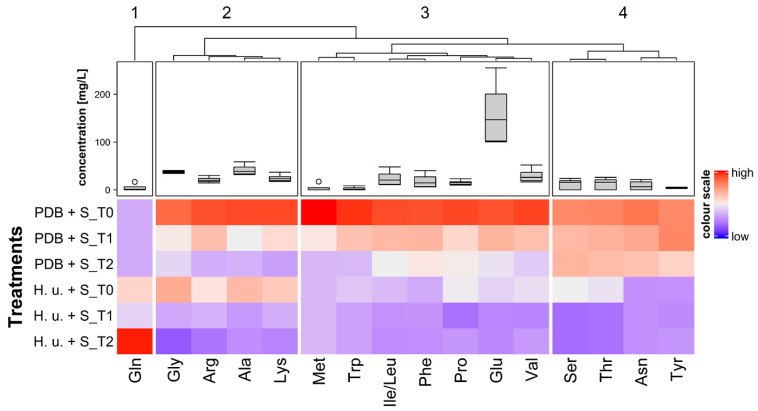
Heatmap of the concentration of amino acids in PDB + S and H. u. + S fermentation broth (T0) and on the surface of leaves treated with PDB + S and H. u. + S collected one day (T1) and one week (T2) after treatment. Three letter codes were used to indicate amino acids. Boxplots show the concentration of amino acids over all six treatments. Clustering of the amino acids was performed using Ward method with Euclidian distance, and the split was based on the k-means algorithm (k = 4).

Overall, 17 amino acids could be measured in all samples (Figure 5). The highest concentrations were found in PDB + S, whereas yeasts consumed amino acids (H. u. + S). As for carbohydrates and sugar alcohols, as soon as the formulation was applied on leaves surface, the concentrations of these compounds started to decrease over time in most of the cases, confirming, as discussed above, that biodegradation probably occurs. Few exceptions were found: the concentrations at the three timepoints of tyrosine in H. u. + S (*p* = 0.879), as well as those of glycine (*p* = 0.078), serine (*p* = 0.293), threonine (*p* = 0.085), and tyrosine (*p* = 0.486), in PDB + S did not change significantly. For glutamine, it was difficult to evaluate differences over time, since the concentrations in samples were found to be extremely variable among replicates, with values increasing over time in the case of H. u. + S. According to Spitaler et al. [12], glutamic acid was the most abundant amino acid present in PDB, as well as in yeast fermentation broth. Concentrations of 143 mg/L and of 255 mg/L of glutamic acids were found in H. u. + S fermentation broth and in PDB + S culture broth, respectively. This compound was reported as phagostimulant for *Drosophila* flies [39]. The availability of glutamic acid on leaves’ surface up to seven days after treatment can be associated to the stimulation of the ingestion of the formulation by SWD. Although a reduction or loss over time of some essential amino acids for SWD, including methionine, threonine, and tryptophan, was observed in H. u. + S, the yeast-based attract-and-kill formulation was found to have a strong effect on SWD survival up to one week after treatment. This may indicate either that the low amount of these compounds does not influence the feeding acceptance of a food source for SWD flies, or that their lack is compensated by the biosynthesis of macromolecules by yeasts.

### 3.5. Organic Acids

**Figure 6:**
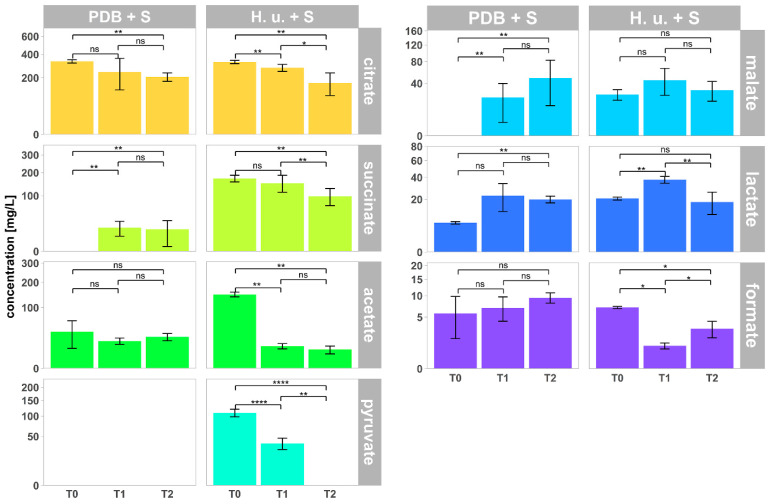
Concentration of organic acids (mean ± SD; *n* = 5) in the culture broth PDB + S (T0), in the fermentation broth H. u. + S (T0) and on the surface of leaves treated with PDB + S and H. u. + S collected one day (T1) and one week (T2) after treatment. Pyruvate was not detected at any of the timepoints in PDB + S. Succinate and malate were not detected at T0 in PDB + S. Asterisks indicate significant differences between timepoints for each treatment (*p* < 0.05). Not significant differences are reported, as well (ns). <0.05 = *; ≤0.01 = **; ≤0.0001 = ****.

The concentrations of seven organic acids in the samples analyzed are reported in Figure 6. As expected, the amounts at T0 of succinate, acetate, pyruvate, and malate, which are products of the yeast metabolism, were much higher in H. u. + S compared to PDB + S. The concentrations of citrate and formate at T0 were instead similar between the two. Citrate was the most abundant organic acid with concentrations of 328 mg/L and 334 mg/L in *H. uvarum* fermentation broth and PDB culture broth, respectively. As sugar alcohols, these compounds constitute a product of the yeast metabolism. A decreasing trend in the concentrations of most of the organic acids was observed, with few exceptions. Time had no influence on malate in H. u. + S (*p* = 0.264) and on formate (*p* = 0.170) and acetate (*p* = 0.403) in PDB + S. The decrease or increase of the concentrations of organic acids can again be a result of the biodegradation. In addition, based on the chemical characteristics of acetate, it is expected that, after drying on the leaf surface, this compound would be mainly present as a vapor in the ambient atmosphere [32], as confirmed by the highly significant reduction of its concentration at T1 in H. u. + S. The high volatility of formate too [40] resulted in a reduction of its concentration on the surface of leaves already one day after treatment with H. u. + S. The rapid degradation of pyruvate already at T1 after treatment with H. u. + S can be explained as a result of the photolysis in presence of sunlight [41].

Previous studies showed that *Drosophila* flies tend to reject too acidic food and to have adverse responses to carboxylic acids, including acetic acid and citric acid [42,43,44]. In addition, sweet perceiving neurons were found to be inhibited by acid taste. However, an increase in sugar concentration allowed to overcome food rejection [42]. Therefore, an appropriate combination of sugar and acid concentrations is crucial to favor the acceptance of a food source by *Drosophila* flies.

### 3.6. VOCs

**Figure 7:**
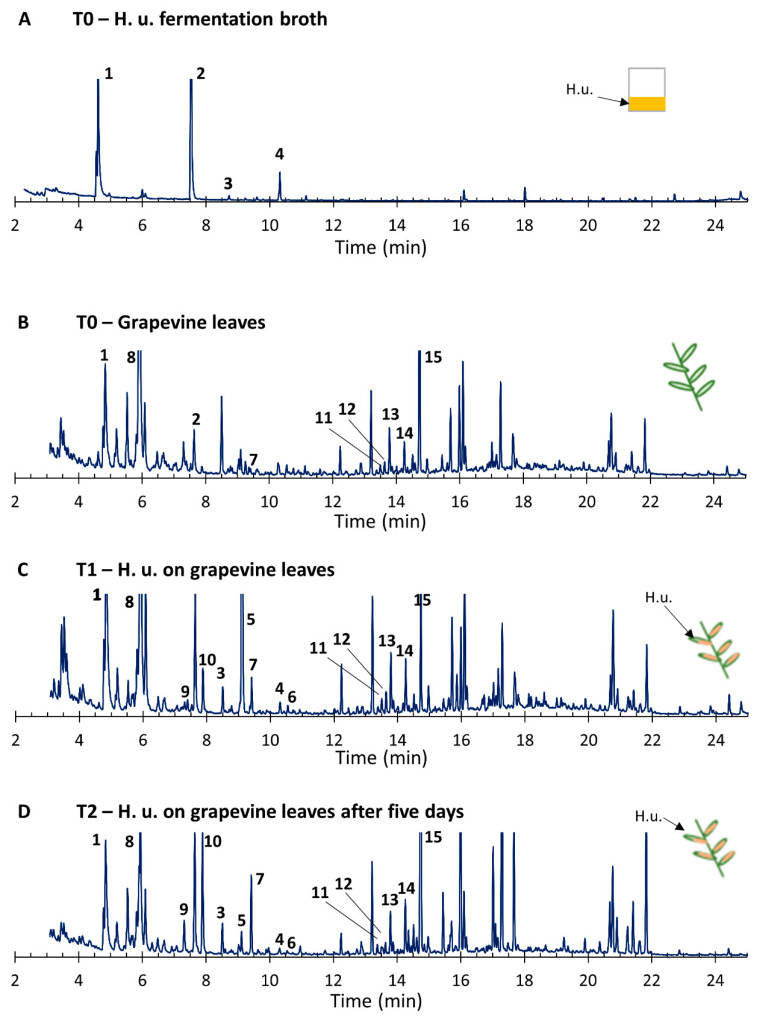
Chromatogram from headspace extracts of H. u. fermentation broth (**A**), grapevine leaves (**B**), grapevine leaves with H. u. 30 min after application (**C**), grapevine leaves with H. u. 5 days after application (**D**). All headspaces were collected with close loop stripping analysis (CLSA) for 3 h. Chemicals identified from H. u. are reported: 1. benzaldehyde; 2. 2-phenylethanol; 3. octanoic acid; 4. 2-phenylethyl acetate; 5. methyl salicylate; 6. indole; 7. (*Z*)-3-hexenyl butyrate; 8. 1,8-cineole; 9. linalool; 10. (*E*)-4,8-dimethylnona-1,3,7-triene (DMNT); 11. beta-caryophyllene; 12. humulene; 13. germacrene D; 14. trans-alpha-bergamotene; 15. (*E,E*)- alpha–farnesene. All graphs are on the same scale showing the total ion chromatogram peaks in time.

The VOCs composition of headspaces from *H. uvarum* culture and *H. uvarum*-treated grapevine leaves was assessed (Figure 7 and Table 1). The yeast culture alone released mostly benzaldehyde and 2-phenylethanol. The latter was also found in the headspace of grapevine leaves, along with benzaldehyde, (*Z*)-3-hexenyl butyrate, beta-caryophyllene, trans-α-bergamotene, (*E,E*)-alpha-farnesene, and VOCs known to be emitted by grapes, such as germacrene D [45]; by grapevines, like humulene [46]; and by other plants, like 1,8-cineole [47], the latter being the most abundant compound released by the non-treated grapevine leaves. After application of *H. uvarum* on the leaves, the volatile profiles changed. The two main compounds detected in *H. uvarum* (benzaldehyde, 2-phenylethanol) were significantly more abundant in the headspace from treated leaves collected 30 min after treatment (T1—VOCs) and after five days (T2—VOCs). In addition, the release of compounds not detected in the grapevine leaves headspaces was induced just after the treatment with *H. uvarum*, including octanoic acid, 2-phenylethyl acetate, methyl salicylate, and (*E*)-4,8-dimethylnona-1,3,7-triene, and were still released after five days but in lower amounts. On the contrary, the compounds indole, linalool (unidentified isomer), and (*E,E*)-alpha-farnesene release were significantly increased after treatment and five days later. The latter was the most abundant VOC released by the treated grapevine leaves five days after application. In total, one aldehyde, one alcohol, one acetate, one acid, one green leaf volatile, two aromatic compounds, and eight terpenes were the characterized volatiles. Most of the terpenes detected in the volatile profiles also showed significant difference among non-treated and *H. uvarum*-treated grapevine leaves.

## 4. Conclusions

Besides being more attractive to SWD adults and larvae compared to other yeast species [20,21,48], *H. uvarum* has been reported to have a beneficial effects in the diet of SWD larvae and to promote the ingestion by SWD adults [12,18]. Based on the aforementioned findings, in the present work, potted grape plants in a greenhouse were treated with an attract-and-kill formulation based on *H. uvarum* fermentation broth containing spinosad. The efficacy and persistence over time of the formulation were evaluated. The addition of the selected yeast to the insecticide resulted in increased mortality of SWD flies and reduced egg-laying. The effect on the survival of SWD was stronger one day after treatment, but the formulation was still effective after one week. Since mortality of SWD flies can be related to attractiveness or feeding stimulation towards specific components of the formulation, potential phagostimulants and attractants were analyzed, in order to determine whether changes in their composition were correlated with the efficacy of the formulation. Several non-volatile compounds, including carbohydrates, sugar alcohols, amino acids, and organic acids, were found in the formulation, the concentrations of which generally decreased over time. Many of these compounds are reported as feeding stimulants for SWD flies, indicating that it is worth investigating the chemical composition of SWD food to improve attract-and-kill control strategies. Numerous VOCs emitted by yeasts and induced in the plant after the treatment were detected, and changes in the VOCs profile over time were observed. Further electrophysiological and behavioral studies may be helpful for the optimization of an effective attract and kill formulation.

## Figures and Tables

**Table 1:** Total Ion Chromatogram (TIC) peak area of volatile organic compounds (VOCs) from yeast-treated and non-treated grapevine leaves collected by Closed Loop Stripping Analysis (CLSA) (*n* = 6). Average amounts of VOCs measured in TIC ^†^ are indicated. The following abbreviations are used: Linear Retention Index (LRI), Green Leaf Volatiles (GLVs), (E)-4,8-dimethylnona-1,3,7-triene (DMNT), not detected (nd). Significant differences (*p* < 0.05) are reported using different letters and asterisks (≥0.05 = ns; <0.05 = *; ≤0.01 = **; ≤0.001 = ***).

No	Compound	LRI ^ꓯ^ on HP-5MS	Reference LRI	Grapevine Leaves (T0)	Grapevine Leaves + *H. uvarum* (T1)	Grapevine Leaves + *H. uvarum* (T2)	Significance (ANOVA *p* Value, df = 2, 15)
*ALDEHYDES* 1	Benzaldehyde ^α^	961	965	0.33 ± 0.28 ^a^	2.85 ± 1.35 ^b^	1.20 ± 1.07 ^ab^	F = 8.039, 0.004 **
*ALCOHOLS* 2	2-phenylethanol ^α^	1114	1116	0.18 ± 0.20 ^a^	1.09 ± 0.45 ^b^	0.48 ± 0.36 ^a^	F = 8.648, 0.004 **
*ACIDS* 3	octanoic acid ^α^	1171	1175	nd ^a^	0.70 ± 0.30 ^b^	0.53 ± 0.39 ^b^	F = 8.431, 0.003 **
*ACETATES* 4	2-phenylethyl acetate ^α^	1258	1265	nd ^a^	1.48 ± 0.60 ^b^	0.96 ± 0.63 ^b^	F = 11.27, 0.001 **
*AROMATICS*							
5	methyl salicylate ^α^	1193	1190	nd ^a^	12.73 ± 9.00 ^b^	2.25 ± 2.17 ^a^	F = 14.45, *p* < 0.001 ***
6	Indole ^α^	1291	1288	nd ^a^	0.57 ± 0.24 ^ab^	1.82 ± 1.85 ^b^	F = 3.75, 0.047 *
*GLVs* 7	(*Z*)-3-hexenyl butyrate ^α^	1188	1180	0.06 ± 0.08	2.63 ± 2.39	3.87 ± 4.99	F = 1.846, ns
*TERPENES*							
8	1,8-cineole	1030	1030	7.80 ± 4.37	24.95 ± 14.86	15.98 ± 12.13	F = 2.851, ns
9	Linalool ^α^	1103	1101	nd ^a^	1.53 ± 0.88 ^ab^	2.79 ± 1.54 ^b^	F = 9.299, 0.002 **
10	DMNT ^α^	1117	1105	nd ^a^	9.07 ± 4.70 ^b^	7.51 ± 5.32 ^b^	F = 6.996, 0.007 **
11	beta-caryophyllene ^α^	1418	1418	0.20 ± 0.15 ^a^	8.90 ± 3.83 ^b^	5.80 ± 5.39 ^ab^	F = 6.659, 0.008 **
12	Humulene ^α^	1452	1440	0.29 ± 0.11 ^a^	5.11 ± 1.94 ^b^	3.49 ± 2.66 ^b^	F = 8.334, 0.004 **
13	germacrene D ^α^	1480	1480	0.80 ± 0.46 ^a^	4.61 ± 2.08 ^b^	3.35 ± 2.41 ^b^	F = 5.478, 0.016 *
14	trans-alpha-bergamotene	1495	1496	0.15 ± 0.22	1.99 ± 0.62	3.63 ± 3.66	F = 3.284, ns
15	(*E,E*)-alpha-farnesene ^α^	1508	1500	0.13 ± 0.11 ^a^	25.02 ± 13.40 ^a^	136.25 ± 115.62 ^b^	F = 5.816, 0.013 *

^ꓯ^ Linear Retention Indices as calculated from experimental retention times; † The amount in TIC of each compound in the yeast cultures is the mean peak area of six replicates; mean ± standard deviation, divided by 10^6^ (in TIC/3 h) of volatile compounds calculated from six replicates. ^α^ VOCs confirmed by comparison with reference standards.

## Supplementary Materials

The following are available online at https://www.mdpi.com/2075-4450/11/11/810/s1, Table S1: Temperature (°C) and relative humidity (%) registered over the experimental period.

## References

[1] Walsh D.B., Bolda M.P., Goodhue R.E., Dreves A.J., Lee J., Bruck D.J., Walton V.M., O’Neal S.D., Zalom F.G. (2011). *Drosophila suzukii* (Diptera: *Drosophilidae*): Invasive Pest of Ripening Soft Fruit Expanding its Geographic Range and Damage Potential. J. Integr. Pest Manag..

[2] Cini A., Ioriatti C., Anfora G. (2012). A review of the invasion of *Drosophila suzukii* in Europe and a draft research agenda for integrated pest management. Bull. Insectol..

[3] Farnsworth D., Hamby K.A., Bolda M., Goodhue R.E., Williams J.C., Zalom F.G. (2017). Economic analysis of revenue losses and control costs associated with the spotted wing drosophila, *Drosophila suzukii* (Matsumura), in the California raspberry industry. Pest Manag. Sci..

[4] Haviland D.R., Beers E.H. (2012). Chemical Control Programs for *Drosophila suzukii* that Comply with International Limitations on Pesticide Residues for Exported Sweet Cherries. J. Integr. Pest Manag..

[5] Mori B.A., Whitener A.B., Leinweber Y., Revadi S., Beers E.H., Witzgall P., Becher P.G. (2017). Enhanced yeast feeding following mating facilitates control of the invasive fruit pest *Drosophila suzukii*. J. Appl. Ecol..

[6] Noble R., Dobrovin-Pennington A., Phillips A., Cannon M.F.L., Shaw B., Fountain M.T. (2019). Improved insecticidal control of spotted wing drosophila (*Drosophila suzukii*) using yeast and fermented strawberry juice baits. Crop Prot..

[7] Knight A.L., Basoalto E., Yee W., Hilton R., Kurtzman C.P. (2016). Adding yeasts with sugar to increase the number of effective insecticide classes to manage *Drosophila suzukii* (Matsumura) (Diptera: *Drosophilidae*) in cherry. Pest Manag. Sci..

[8] Cha D.H., Adams T., Werle C.T., Sampson B.J., Adamczyk J.J., Rogg H., Landolt P.J. (2014). A four-component synthetic attractant for *Drosophila suzukii* (Diptera: *Drosophilidae*) isolated from fermented bait headspace. Pest. Manag. Sci..

[9] Iglesias L.E., Nyoike T.W., Liburd O.E. (2014). Effect of Trap Design, Bait Type, and Age on Captures of *Drosophila suzukii* (Diptera: *Drosophilidae*) in Berry Crops. J. Econ. Entomol..

[10] Roubos C.R., Gautam B.K., Fanning P.D., Van Timmeren S., Spies J., Liburd O.E., Isaacs R., Curry S., Little B.A., Sial A.A. (2019). Impact of phagostimulants on effectiveness of OMRI-listed insecticides used for control of spotted-wing drosophila (*Drosophila suzukii* Matsumura). J. Appl. Entomol..

[11] Ljungdahl P.O., Daignan-Fornier B. (2012). Regulation of amino acid, nucleotide, and phosphate metabolism in *Saccharomyces cerevisiae*. Genetics.

[12] Spitaler U., Bianchi F., Eisenstecken D., Castellan I., Angeli S., Dordevic N., Robatscher P., Vogel R.F., Koschier E.H., Schmidt S. (2020). Yeast species affects feeding and fitness of *Drosophila suzukii* adults. J. Pest Sci..

[13] Ye M., Yue T., Yuan Y. (2014). Changes in the profle of volatile compounds and amino acids during cider fermentation using dessert variety of apples. Eur. Food Res. Technol..

[14] Callejón R.M., Margulies B., Hirson G.D., Ebeler S.E. (2012). Dynamic changes in volatile compounds during fermentation of Cabernet Sauvignon grapes with and without skins. Am. J. Enol. Vitic..

[15] Cha D.H., Adams T., Rogg H., Landolt P.J. (2012). Identification and Field Evaluation of Fermentation Volatiles from Wine and Vinegar that Mediate Attraction of Spotted Wing Drosophila, *Drosophila suzukii*. J. Chem. Ecol..

[16] Fountain M.T., Bennett J., Cobo-Medina M., Conde Ruiz R., Deakin G., Delgado A., Harrison R., Harrison N. (2018). Alimentary microbes of winter-form *Drosophila suzukii*. Insect Mol. Biol..

[17] Hamby K.A., Hernández A., Boundy-Mills K., Zalom F.G. (2012). Associations of yeasts with spotted-wing Drosophila (*Drosophila suzukii*; Diptera: *Drosophilidae*) in cherries and raspberries. Appl. Environ. Microbiol..

[18] Bellutti N., Gallmetzer A., Innerebner G., Schmidt S., Zelger R., Koschier E.H. (2018). Dietary yeast affects preference and performance in *Drosophila suzukii*. J. Pest Sci..

[19] Lewis M.T., Koivunen E.E., Swett C.L., Hamby K.A. (2019). Associations between *Drosophila suzukii* (Diptera: *Drosophilidae*) and Fungi in Raspberries. Environ. Entomol..

[20] Scheidler N.H., Liu C., Hamby K.A., Zalom F.G., Syed Z. (2015). Volatile codes: Correlation of olfactory signals and reception in *Drosophila*-yeast chemical communication. Sci. Rep..

[21] Lewis M.T., Hamby K.A. (2019). Differential Impacts of Yeasts on Feeding Behavior and Development in Larval *Drosophila suzukii* (Diptera: *Drosophilidae*). Sci. Rep..

[22] Andreazza F., Bernardi D., Baronio C.A., Pasinato J., Nava D.E., Botton M. (2017). Toxicities and effects of insecticidal toxic baits to control *Drosophila suzukii* and *Zaprionus indianus* (Diptera: *Drosophilidae*). Pest Manag. Sci..

[23] Van Den Dool H., Kratz P.D. (1963). A generalization of the retention index system including linear temperature programmed gas-liquid partition chromatography. J. Chromatogr. A.

[24] Team R.C. (2019). R: A Language and Environment for Statistical Computing.

[25] Wickham H. (2016). ggplot2 Elegant Graphics for Data Analysis (Use R!).

[26] Cowles R.S., Rodriguez-Saona C., Holdcraft R., Loeb G.M., Elsensohn J.E., Hesler S.P. (2015). Sucrose Improves Insecticide Activity Against *Drosophila suzukii* (Diptera: *Drosophilidae*). J. Econ. Entomol..

[27] Jaramillo S.L., Mehlferber E., Moore P.J. (2015). Life-history trade-offs under different larval diets in *Drosophila suzukii* (Diptera: *Drosophilidae*). Physiol. Entomol..

[28] Clymans R., Van Kerckvoorde V., Bangels E., Akkermans W., Alhmedi A., De Clercq P., Beliën T., Bylemans D. (2019). Olfactory preference of *Drosophila suzukii* shifts between fruit and fermentation cues over the season: Effects of physiological status. Insects.

[29] Swoboda-Bhattarai K.A., McPhie D.R., Burrack H.J. (2017). Reproductive Status of *Drosophila suzukii* (Diptera: *Drosophilidae*) Females Influences Attraction to Fermentation-Based Baits and Ripe Fruits. J. Econ. Entomol..

[30] Argüelles J.C. (2000). Physiological roles of trehalose in bacteria and yeasts: A comparative analysis. Arch. Microbiol..

[31] Zaunmüller T., Unden G., König H., Unden G., Fröhlich J. (2009). Transport of sugars and sugar alcohols by lactic acid bacteria. Biology of Microorganisms on Grapes, in Must and in Wine.

[32] Wernke M.J., Wexler P. (2014). Glycerol. Encyclopedia of Toxicology.

[33] Biolchini M., Murru E., Anfora G., Loy F., Banni S., Crnjar R., Sollai G. (2017). Fat storage in *Drosophila suzukii* is influenced by different dietary sugars in relation to their palatability. PLoS ONE.

[34] Isono K., Morita H., Kohatsu S., Ueno K., Matsubayashi H., Yamamoto M.T. (2005). Trehalose sensitivity of the gustatory receptor neurons expressing wild-type, mutant and ectopic Gr5a in *Drosophila*. Chem. Senses.

[35] Dahanukar A., Lei Y.T., Kwon J.Y., Carlson J.R. (2007). Two Gr Genes Underlie Sugar Reception in *Drosophila*. Neuron.

[36] Slone J., Daniels J., Amrein H. (2007). Sugar Receptors in *Drosophila*. Curr. Biol..

[37] Wisotsky Z., Medina A., Freeman E., Dahanukar A. (2011). Evolutionary differences in food preference rely on Gr64e, a receptor for glycerol. Nat. Neurosci..

[38] Kim H., Kim H., Kwon J.Y., Seo J.T., Shin D.M., Moon S.J. (2018). *Drosophila* Gr64e mediates fatty acid sensing via the phospholipase C pathway. PLoS Genet..

[39] Yang Z., Huang R., Fu X., Wang G., Qi W., Mao D., Shi Z., Shen W.L., Wang L. (2018). A post-ingestive amino acid sensor promotes food consumption in *Drosophila*. Cell Res..

[40] Salthammer T. (2016). Very volatile organic compounds: An understudied class of indoor air pollutants. Indoor Air.

[41] Grosjean D. (1983). Atmospheric reactions of pyruvic acid. Atmos. Environ..

[42] Charlu S., Wisotsky Z., Medina A., Dahanukar A. (2013). Acid sensing by sweet and bitter taste neurons in *Drosophila melanogaster*. Nat. Commun..

[43] Revadi S., Vitagliano S., Rossi Stacconi M.V., Ramasamy S., Mansourian S., Carlin S., Vrhovsek U., Becher P.G., Mazzoni V., Rota-Stabelli O. (2015). Olfactory responses of *Drosophila suzukii* females to host plant volatiles. Physiol. Entomol..

[44] Liman E.R., Zhang Y.V., Montell C. (2014). Peripheral coding of taste. Neuron.

[45] May B., Lange B.M., Wüst M. (2013). Biosynthesis of sesquiterpenes in grape berry exocarp of Vitis vinifera L.: Evidence for a transport of farnesyl diphosphate precursors from plastids to the cytosol. Phytochemistry.

[46] Chalal M., Winkler J.B., Gourrat K., Trouvelot S., Adrian M., Schnitzler J.P., Jamois F., Daire X. (2015). Sesquiterpene volatile organic compounds (VOCs) are markers of elicitation by sulfated laminarine in grapevine. Front. Plant Sci..

[47] Erland L.A.E., Rheault M.R., Mahmoud S.S. (2015). Insecticidal and oviposition deterrent effects of essential oils and their constituents against the invasive pest *Drosophila suzukii* (Matsumura) (Diptera: *Drosophilidae*). Crop Prot..

[48] Batista M.R.D., Uno F., Chaves R.D., Tidon R., Rosa C.A., Klaczko L.B. (2017). Differential attraction of drosophilids to banana baits inoculated with *Saccharomyces cerevisiae* and *Hanseniaspora uvarum* within a Neotropical forest remnant. PeerJ.

